# Interpretable Machine Learning Prediction of Polyimide Dielectric Constants: A Feature-Engineered Approach with Experimental Validation

**DOI:** 10.3390/polym17121622

**Published:** 2025-06-11

**Authors:** Xiaojie He, Jiachen Wan, Songyang Zhang, Chenggang Zhang, Peng Xiao, Feng Zheng, Qinghua Lu

**Affiliations:** 1 School of Chemical Science and Engineering, Tongji University, Siping Road No. 1239, Shanghai 200092, China; 2 Institute of Micro/Nano Materials and Devices, Ningbo University of Technology, Fenghua Road No. 201, Ningbo 315211, China; 3 State Key Laboratory of Synergistic Chem-Bio Synthesis, School of Chemistry and Chemical Engineering, Shanghai Jiao Tong University, Shanghai 200240, China; 4 State Key Laboratory of Micro-Nano Engineering Science, Shanghai Jiao Tong University, 800 Dongchuan Road, Shanghai 200240, China

**Keywords:** polyimide, machine learning, dielectric constant

## Abstract

Low-dielectric polyimides (PIs) have emerged as essential materials for next-generation microelectronics and communication technologies, yet traditional experimental and theoretical calculation methods for acquiring dielectric constant data face challenges in cost, accuracy, and scalability. This study presents a machine learning (ML) framework that combines polymer domain knowledge with advanced data-driven modeling techniques for accurate prediction of PI dielectric constants at 1 kHz. A dataset of 439 PIs was constructed, and 208 molecular descriptors were derived from SMILES-encoded structures. Through rigorous feature engineering—variance filtering, correlation analysis, and recursive feature elimination—10 key descriptors were identified, capturing electronic and polar interaction, surface area, and structural complexity. Five ML algorithms were evaluated, with Gaussian Process Regression (GPR) achieving superior predictive accuracy (test set: R^2^ = 0.90, RMSE = 0.10). Shapley additive explanations (SHAP) analysis quantifies the contribution of molecular descriptors to PI dielectric constants. By means of SHAP values, it discloses the positive or negative impacts of descriptors on the predictions. Three novel PIs were synthesized for experimental validation, showing strong agreement between predicted and measured dielectric constants (mean percentage deviation: 2.24%). The model demonstrates robust predictions for other structurally similar polymers but reveals a 40% accuracy reduction (R^2^ = 0.60) in 10 GHz cross-frequency predictions, emphasizing the requirement for multi-frequency training datasets to enhance model generalizability. This work advances the research paradigm of polymer dielectric materials and provides a pathway for the rational design of materials guided by machine learning.

## 1. Introduction

Engineering plastics low-dielectric materials are crucial for reducing signal delay, minimizing cross talk, and improving the performance of high-frequency electronics [1,2,3]. Among high-performance polymers, polyimides (PIs) are particularly notable for their exceptional thermal stability, mechanical strength, and chemical resistance. However, conventional PIs often exhibit high dielectric constants, limiting their applications in advanced electronics. This limitation has driven substantial research efforts toward developing low-dielectric-constant alternatives, establishing it as a key objective in contemporary materials science and engineering [4].

Experimental measurements are the primary approach for determining the dielectric constants of PIs; these methods are often time-intensive, expensive, and prone to variability due to inconsistencies in experimental conditions and sample preparation [5,6]. Current computational approaches for dielectric constant prediction, notably quantum chemical calculations and molecular dynamics (MD) simulations [7,8,9], still face distinct challenges. Methods like Density Functional Perturbation Theory (DFPT) are restricted to small systems (<50 atoms) to maintain computational feasibility, and even then neglect dipolar contributions critical for polymer dielectrics [10]. In contrast, classical force fields and MD simulations are computationally efficient but often sacrifice reliability and quantitative accuracy. This discrepancy between computational predictions and experimental results underscores the limitations of relying exclusively on simulation data [11].

Artificial intelligence (AI)-based methods have significantly advanced materials development, enabling predictions of thermal stability [12,13,14], mechanical strength [15,16], and optical transparency [17,18]. Machine learning (ML) has emerged as a powerful tool for utilizing large datasets to predict polymer dielectric properties. However, challenges remain in applying ML to low-dielectric materials, particularly in model interpretability, data quality, and the integration of domain-specific knowledge [19,20].

To alleviate these concerns, a ML framework was developed to predict the dielectric constant of PIs. This methodology combines data science with chemical insights through dataset construction, key molecular descriptor selection, and advanced machine learning algorithms to establish structure–dielectric relationships. The model successfully predicted dielectric constants for three novel PI structures at 1 kHz, demonstrating excellent agreement with experimental values. Furthermore, machine learning models exhibit limited accuracy in predicting the dielectric constant across different frequencies for PIs, with an R^2^ of 0.60, emphasizing the need for data augmentation at various frequencies. High structural similarity, indicated by a Tanimoto similarity score greater than 0.15, improves prediction accuracy for other polymers.

**Figure 1:**
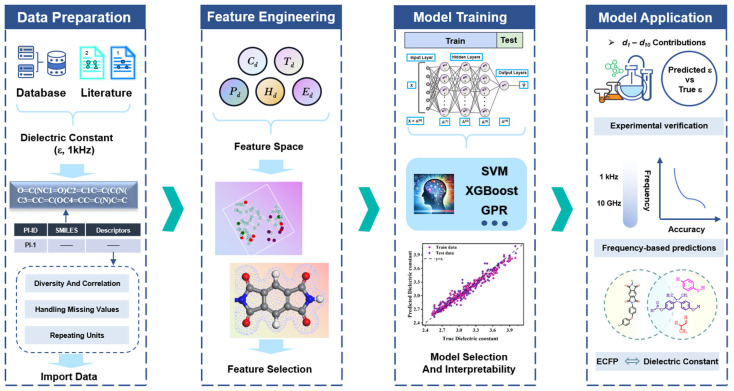
Machine learning workflow for developing dielectric constant models of PIs, including data preparation, feature engineering, model training and model application.

Figure 1 outlines the workflow for the machine learning-guided dielectric constant prediction of polymer materials. The dataset includes 439 PIs with dielectric constant values reported in the literature at 1 kHz. Initially, the chemical structures of the PIs were converted into SMILES format, and 208 descriptors were extracted using the RDKit package in Python 3.9. Through variance filtering, correlation analysis, and feature importance evaluation, 10 descriptors were identified as representations of structural information. The selected 10 descriptors served as input parameters for training machine learning models using five algorithms: extreme gradient boosting (XGBoost), Gaussian process regression (GPR), random forest (RF), artificial neural network (ANN), and support vector machine (SVM). The trained models predicted dielectric constant at 1 kHz for the PI samples in the test set. Subsequent SHAP analysis revealed descriptor–dielectric relationship. Complete computational details are provided in Section 2.

This study promotes the development of machine learning applied to low dielectric materials, including descriptor selection, model building, and model interpretability. It also clarifies how the structural information of PIs affects dielectric properties, providing theoretical guidance for the design of low dielectric polymers.

## 2. Methods

**Descriptor generation and selection.** The PI repeating unit structures were converted to SMILES format, and 208 molecular descriptors were extracted using the RDKit cheminformatics toolkit (see Note S1 for details) [21]. A systematic approach for feature selection was employed. First, variance thresholding was applied [22] with a variance of less than 0.01. From the remaining descriptors, feature importance was assessed using the random forest algorithm. Subsequently, recursive feature elimination (RFE) was utilized to identify the optimal set of descriptors. RFE is an effective feature selection technique that iteratively eliminates the least important features based on model performance until the optimal subset of features is reached [23]. This selection process also considered the correlation of the descriptors with the dielectric constant. Ultimately, some descriptors were finalized for subsequent machine learning modeling. Additionally, due to the frequency dependence of dielectric constants [24], all samples in the dataset were measured at 1 kHz, leading to the exclusion of frequency as a descriptor to maintain data consistency.

**Figure 2:**
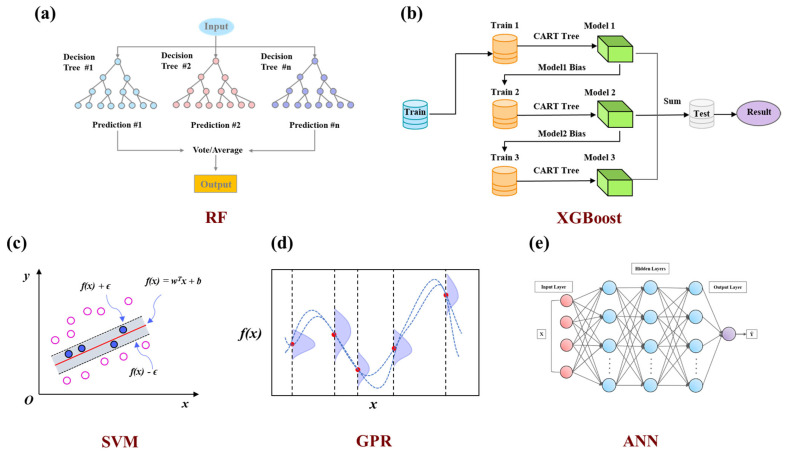
Comparison of machine learning algorithms for predicting PI dielectric constant: (**a**) RF. (**b**) XGBoost. (**c**) SVM. (**d**) GPR. (**e**) ANN.

**Machine learning strategy.** Five machine learning algorithms (Figure 2a–e) were evaluated for dielectric constant prediction in PIs, representing diverse modeling approaches: ensemble methods (RF and XGBoost) [25], kernel-based learning (SVM) [26], probabilistic modeling (GPR), and deep learning (ANN) [27]. Since regression algorithms can exhibit varying predictive performances on the same dataset, comparing multiple algorithms helps identify the most accurate and robust model. Model training was implemented using the Scikit-learn library [28], the dataset was split into 80% for training and 20% for testing. Model performance was assessed using R-Square (R^2^), Root Mean Squared Error (RMSE), and Mean Absolute Error (MAE) by averaging the statistical runs of 30 random training and test splits.

**Evaluation of structural similarity.** Structural similarity analysis was conducted to assess model transferability to other polymers. PMDA-ODA, a representative PI structure, and 12 additional polymers were represented by SMILES notation and converted to Extended-Connectivity Fingerprints (ECFPs) [29]. Tanimoto similarity scores between PMDA-ODA and the 12 polymers were calculated to quantify their structural relationships (see Note S2 for calculation details). The Tanimoto similarity score [30] is defined as Equation (1):

(1) $$T_{st}=\frac{\sum_{k=1}^{n}P_{sk}\cdot P_{tk}}{\sum_{k=1}^{n}P_{sk}^{2}+\sum_{k=1}^{n}P_{tk}^{2}-\sum_{k=1}^{n}P_{sk}\cdot P_{tk}}$$

The Tanimoto similarity score measures the similarity between two molecular fingerprint vectors, *P_s_* and *P_t_*, where *P_sk_* and *P_tk_* represent the *k*-th components of the fingerprints for molecules *s* and *t*, respectively. The numerator $\sum_{k=1}^{n}P_{sk}\cdot P_{tk}$ calculates the shared features between *s* and *t*. The denominator represents the combined features without double-counting shared features. The resulting coefficient ranges from 0 to 1, where 1 indicates identical fingerprints, and 0 indicates no shared features.

**Descriptor correlation analysis.** Model training was the Pearson correlation coefficient *r*, which quantifies the strength and direction of a linear relationship between two variables, calculated as Equation (2)

(2) $$r=\frac{\sum \left(x_{i}-\bar{x}\right)\left(y_{i}-\bar{y}\right)}{\sqrt{\sum {\left(x_{i}-\bar{x}\right)}^{2}}\cdot \sqrt{\sum {\left(y_{i}-\bar{y}\right)}^{2}}}$$

where *x* and *y* are one-dimensional arrays of the same length, *x_i_* and *y_i_* represent individual values within these arrays, and $\bar{x}$ and $\bar{y}$ are the respective sample means. The coefficient *r* ranges from −1 to 1, with values closer to ±1 indicating a stronger linear relationship and values near 0 indicating little or no linear association.

## 3. Results and Discussion

**Figure 3:**
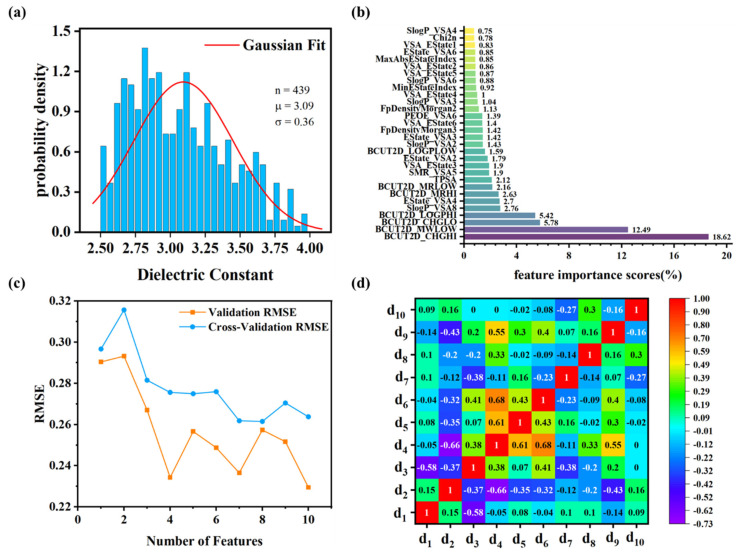
The dielectric constant values and selected descriptors of PIs. (**a**) Dielectric constant data distribution, where *n* is the data sample size, *μ* is the mean value, and *σ* is the standard deviation. (**b**) The feature importance scores of descriptors derived from the random forest algorithm, highlighting the top 30 descriptors. (**c**) RMSE trends across validation and cross-validation during recursive feature elimination. (**d**) Correlation coefficients between the top 10 descriptors.

**Dataset and polymer descriptors.** The dataset was compiled from previously published literature (with URLs listed in Table S1). The dielectric constants span from 2.52 to 3.96, with a mean (μ) of 3.09 and a standard deviation (σ) of 0.36 (Figure 3a). Although the distribution is approximately normal, it exhibits slight skewness. To ensure consistency in model inputs, data normalization was applied during the machine learning workflow. Following variance screening, 68 descriptors with a fluctuation variance below 0.01 were removed. Among the remaining 140 descriptors, four pairs were identified with identical values: MaxEStateIndex and MaxAbsEStateIndex, NumAromaticCarbocycles and fr_benzene, fr_Ar_NH and fr_Nhpyrrole, and fr_C_O and fr_C_O_noCOO. To reduce redundancy, MaxEStateIndex, fr_benzene, fr_Ar_NH, and fr_C_O were excluded, as the remaining descriptors offered clearer, more distinct chemical insights.

Feature importance scores for descriptors were calculated (Table S2). Figure 3b presents descriptors with feature importance scores of 0.75% or higher, spanning categories such as molecular electrostatic, topological, and molecular surface area descriptors. RFE was utilized to determine the optimal feature subset size. As illustrated in Figure 3c, the validation and cross-validation RMSE values reach their minima at 10 features, representing an optimal trade-off between model accuracy and complexity. The 10 descriptors were selected based on their importance scores and diversity in type, with correlation analysis confirming their independence. The heat map (Figure 3d) demonstrates low-to-moderate pairwise correlations, indicating minimal descriptor redundancy. The final 10 descriptors (Table 1) form an optimized feature set that effectively captures essential chemical information for accurate PI property prediction.

**Figure 4:**
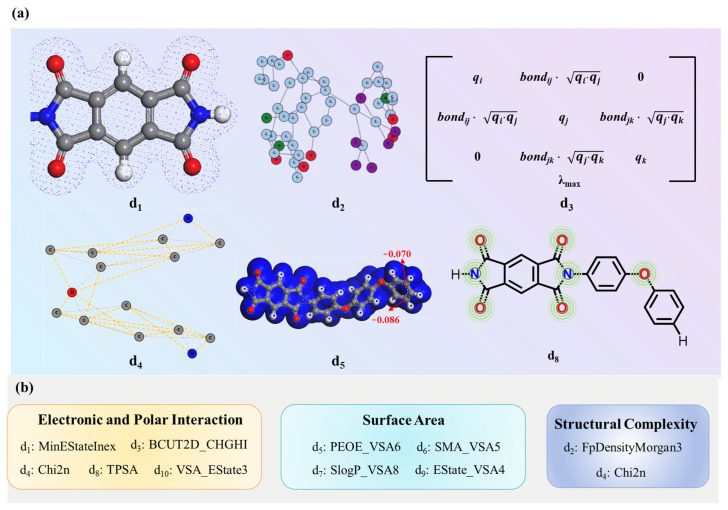
(**a**) The chemical significance of selected descriptors. (**b**) Classification of the ten molecular descriptors.

**Effect of selected descriptors on dielectric constant.** Ten key descriptors were identified. Descriptors MinEStateIndex (d_1_), BCUT12D_CHGHI (d_3_), Chi2n (d_4_), TPSA (d_8_), and VSA_EState3 (d_10_) collectively characterize electronic and polar interaction that influence dielectric behavior. MinEStateIndex (d_1_) identifies low electronic state atoms in polar groups, particularly electronegative elements (O, N), which enhance molecular polarity and polarizability under alternating electric fields [31]. BCUT2D_CHGHI (d_3_) measures localized charge density, where high charge concentration regions typically reduce polarizability, showing an inverse relationship with the dielectric constant. Chi2n (d_4_) reflects the degree of charge separation and local polarity distribution within a molecule. A higher Chi2n (d_4_) value indicates the presence of a greater number of second-order adjacent atom pairs exhibiting significant charge disparities, which may result in strongly polar regions within the molecular structure. TPSA (d_8_) measures the total polar surface area, which highlights electronegative regions (Figure 4a) that enhance electric field response [32]. VSA_EState3 (d_10_) represents the summation of electrotopological state (E-state) indices for atoms whose van der Waals surface area (VSA) values fall within the range of 5.00–5.41 Å^2^. This descriptor quantifies the electron-surface coupling effects in weakly polar regions of the molecule. Both descriptors (VSA_EState3 (d_10_) and MinEStateIndex (d_1_)) are intrinsically linked to the E-State Index framework, as they quantify the electronic characteristics of atoms within a molecule. Therefore, the visualization strategy for VSA_EState3 (d_10_) can adopt methodologies analogous to those used for MinEStateIndex (d_1_).

FpDensityMorgan3 (d_2_) and Chi2n (d_4_) characterize structural complexity [33]; a higher FpDensityMorgan3 (d_2_) value often indicates increased structural complexity due to a greater presence of aromatic or heterocyclic rings and branching [34,35]. Chi2n (d_4_) quantifies molecular connectivity, where higher values indicate increased branching.

Descriptors PEOE_VSA6 (d_5_), SMR_VSA5 (d_6_), SlogP_VSA8 (d_7_), and EState_VSA4 (d_9_) assess molecular surface area. As shown in Figure 4a, the blue region represents the VSA values across all atoms in the molecule; PEOE_VSA6 (d_5_) calculates the VSA values of all atoms whose partial lie between −0.10 and −0.05. SMR_VSA5 (d_6_) identifies electron-dense regions that hinder charge redistribution and polarization response. SMR_VSA5 (d_6_), SlogP_VSA8 (d_7_), and EState_VSA4 (d_9_) quantify the summation of VSA values for atoms within specific ranges of molar refractivity (SMR), octanol–water partition coefficient (logP), and E-state indices, respectively. While these descriptors are fundamentally tied to VSA contributions, their visualization is omitted from Figure 4a to avoid redundancy, as their graphical representation would follow analogous methodologies to the VSA_EState3 (d_10_) and MinEStateIndex (d_1_) mappings already illustrated. Calculation methods of some descriptors are detailed in Table S3. These descriptors comprehensively characterize how electronic and polar interaction, structural complexity, and surface area govern PI dielectric performance (Figure 4b).

**Figure 5:**
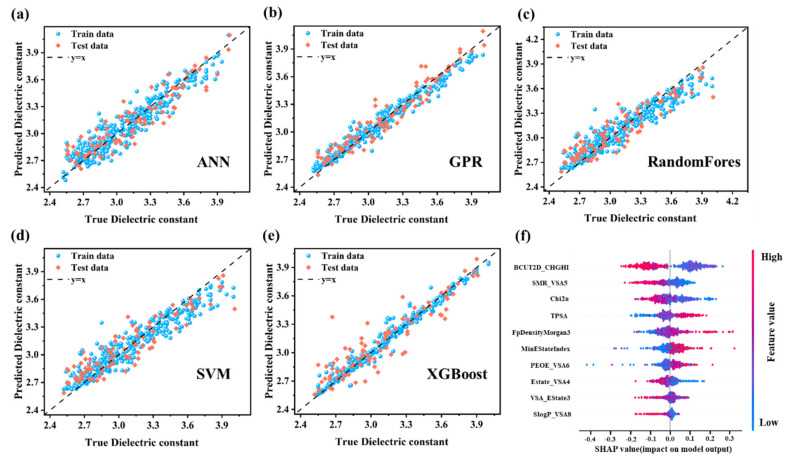
Performance evaluation of machine learning regression models for PI dielectric constant prediction: (**a**) ANN, (**b**) GPR, (**c**) RF, (**d**) SVM, and (**e**) XGBoost. (**f**) SHAP value distribution of selected descriptors.

**Performance Evaluation of ML Models.** Five machine learning algorithms were utilized (Figure 5a–e) to build models based on the ten selected descriptors. Model performance was evaluated by comparing experimental and predicted dielectric constants (Table S4). The GPR model exhibited superior predictive accuracy, achieving the lowest RMSE values (0.07 for training, 0.10 for test) and highest R^2^ values (0.96 for training, 0.90 for test), and demonstrating excellent generalization across the data distribution. These results highlight GPR’s effectiveness in modeling the nonlinear dielectric behavior of PIs, establishing it as an optimal approach for high-precision property prediction. The ANN model demonstrated robust performance, achieving R^2^ values of 0.90 (training) and 0.85 (test), with corresponding RMSE values of 0.11 and 0.14. Its neural network architecture enables effective capture of complex nonlinear patterns, making it particularly suitable for modeling intricate dependencies in PI dielectric properties. While ANN proves to be a viable approach for handling sophisticated nonlinearities, its predictive accuracy on the test set was marginally lower compared to GPR. The XGBoost and RF models demonstrated moderate predictive performance, each with distinct strengths. XGBoost, utilizing gradient boosting to iteratively correct errors, achieved an R^2^ of 0.97 for the training set and 0.84 for the test set, with RMSE values of 0.10 and 0.15, respectively. Although the model exhibits high training accuracy, indicating strong data fitting, its relatively lower test performance suggests limited generalization to unseen data. The RF model, a bagging ensemble method, achieved an R^2^ of 0.89 on the training set and 0.83 on the test set, with RMSE values of 0.11 and 0.15. This performance implies that RF captured certain structural features related to dielectric behavior, but it may lack the precision required to fully represent complex molecular interactions, especially considering the diversity within the dataset. Although the SVM model demonstrated reasonable accuracy, its performance was inferior to that of GPR and ANN, achieving R^2^ values of 0.87 (training) and 0.82 (test), with RMSE values of 0.13 and 0.16, respectively. This lower performance likely reflects the sensitivity of SVM to complex data distributions and susceptibility to noise in the dataset.

While machine learning models demonstrate strong predictive accuracy for PI dielectric constants, their practical applicability and reliability heavily depend on model interpretability [36]. To address this critical aspect, we implemented the XGBoost algorithm coupled with Shapley additive explanations (SHAP), a game theory-based approach that quantifies individual feature contributions to model predictions [37]. The integration is superior to other models, including TreeExplainer’s deterministic Shapley value calculation, high computational efficiency, and hierarchical feature interaction mapping [38,39]. Figure 5f illustrates two key aspects of the model’s dielectric constant predictions: (1) the critical molecular descriptors influencing the predictions and (2) their directional impacts on the results (positive or negative). Descriptors are ranked vertically in descending order of importance, where BCUT2D_CHGHI exhibits the strongest predictive influence, followed by SMR_VSA5, while SlogP_VSA8 contributes minimally. Each data point represents a sample’s descriptor value, with red and blue colors corresponding to higher and lower descriptor magnitudes, respectively. The horizontal SHAP value axis quantifies directional effects: positive SHAP values (rightward points) increase dielectric constant prediction values, whereas negative values (leftward points) decrease the predictions. Taking TPSA as an example, the majority of red points cluster in the positive SHAP region, while blue points dominate the negative zone. This indicates higher TPSA values correlate with increased dielectric constant predictions, whereas lower TPSA values reduce predictions. Such behavior aligns with the fundamental relationship between molecular polar surface area and dielectric properties, reinforcing the physicochemical interpretability of the model.

**Figure 6:**
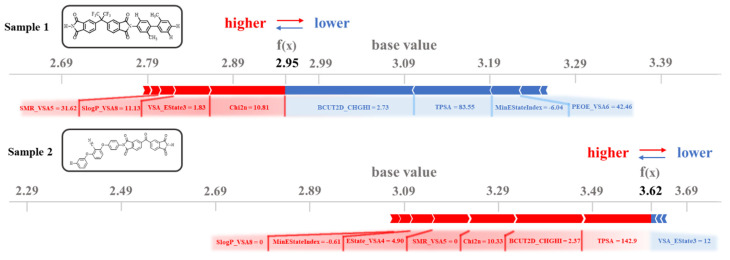
Descriptor impact analysis on dielectric constant prediction for two representative PI structures.

Figure 6, generated via Python code, presents a Force Plot that visually compares the impacts of descriptors on dielectric constant predictions for two distinct samples. The base value (3.09), derived as the mean of 439 collected PI samples, serves as the model’s initial reference point. The *f*(*x*) values denote the actual predicted dielectric constants for individual samples. These predictions are calculated relative to the baseline, with descriptors enhancing the dielectric constant highlighted in red and those reducing it in blue. The numerical annotations adjacent to each descriptor indicate their specific molecular values. For sample 1 (predicted value: 2.95), the descriptors predominantly responsible for reducing the dielectric constant, such as BCUT2D_CHGHI, TPSA, MinEStateIndex, and PEOE_VSA6, exhibit a synergistic interaction, collectively driving the prediction below the baseline.

Notably, while the descriptors like SMR_VSA5, SlogP_VSA8, VSA_Estate3, and Chi2n partially counterbalance this trend by increasing the dielectric constant, their contributions remain insufficient to reverse the overall downward prediction. For the other two descriptors, EState_VSA4 and FpDensityMorgan3, the contribution to the dielectric constant prediction for this sample is small. In contrast, sample 2, with predicted value of 3.62, demonstrates a significant elevation above the base value, driven by seven dominant descriptors that amplify the dielectric constant: TPSA, BCUT2D_CHGHI, Chi2n, SMR_VSA5, Estate_VSA4, MinEStateIndex, and SlogP_VSA8. Of particular interest is the dual regulatory behavior of BCUT2D_CHGHI: while suppressing dielectric constant in sample 1, it enhances the prediction in sample 2. This reversal highlights the strong dependence of descriptors on molecular bonding configurations and its mechanistic link to spatial reorientation of molecular dipoles. This interpretability framework not only validates the role of TPSA in classical dielectric theory but also uncovers critical contributions from counterintuitive descriptors such as Chi2n and BCUT2D_CHGHI. These findings establish a robust descriptor foundation for the rational multiscale design of dielectric materials. By strategically leveraging descriptor synergies, this approach holds promise for overcoming the limitations of empirical trial-and-error methodologies.

**Figure 7:**
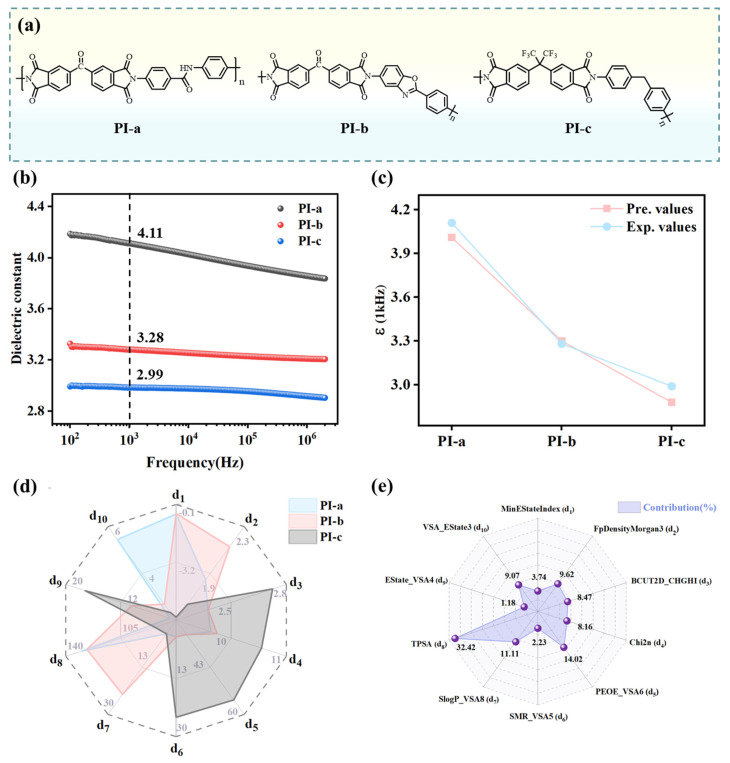
(**a**) Structures of the three PIs. (**b**) Frequency-dependent dielectric constants of PIs. (**c**) Comparison between experimental and predicted dielectric constants. (**d**) Calculated descriptor values for PI-a, PI-b, and PI-c. (**e**) Calculated ten descriptor contributions.

**Experimental validation and extension of the dielectrics constant prediction model.** Based on monomer synthesis feasibility, membrane preparation practicality, and chemical structure representativeness, three PIs—**PI-a**, **PI-b**, and **PI-c**—were selected as optimal candidates for experimental validation (Figure 7a). These PIs were successfully synthesized (Note S3 for experimental details) and their dielectric constants were determined, as shown in Figure 7b. At 1 kHz, **PI-a** demonstrated the highest dielectric constant (4.11), whereas **PI-c** exhibited the lowest (2.99). The GPR model was utilized to predict dielectric constants of the three PIs to further validate its predictive capability, yielding values of 4.01 (**PI-a**), 3.30 (**PI-b**), and 2.88 (**PI-c**) at 1 kHz (Figure 7c). The GPR model-predicted dielectric constants showed a slight underestimation compared to the experimental values, with an average percentage deviation of 2.24% (Table S5). Notably, both values exhibited consistent trends within acceptable experimental error margins.

The differences in dielectric constants of the three PIs can be explained from the perspectives of both molecular structures and the descriptors. Structurally, **PI-a** and **PI-b** incorporate polar functional groups (e.g., carbonyl) with broader polar region distributions, leading to larger dipole moments. This enhances dipolar polarization under an electric field, thereby increasing their dielectric constants. The CF_3_ groups in **PI-c** exhibit a strong electron-withdrawing effect, which effectively reduces the polarizability. Furthermore, the incorporation of the sterically bulky CF_3_ groups disrupts the ordered packing of polymer chains, resulting in increased free volume. This structural modification synergistically contributes to lowering the dielectric constant.

Moreover, ten descriptors were evaluated for their contributions to the three PIs using sensitivity analysis (Table S6). This involved systematically perturbing each descriptor value while monitoring corresponding shifts in GPR model predictions to establish parameter influence. As shown in Figure 7d, **PI-a** and **PI-b** demonstrate significantly higher TPSA (d_8_) values than **PI-c**, reflecting their broader distribution of polar regions, a critical factor for enhanced dielectric properties. This observation is further supported by the descriptor contribution analysis (Figure 7e), where TPSA (d_8_) exhibits the highest contribution (32.42%). The MinEStateIndex (d_1_) values for **PI-a** and **PI-b** were calculated at −0.61 and −0.57, respectively, while **PI-c** exhibited a MinEStateIndex of −6.03. A smaller (more negative) MinEStateIndex value indicates the presence of stronger electron-withdrawing groups within the molecular structure. This substantial reduction in **PI-c** arises from the strong electron-withdrawing CF_3_ group, which induces extreme electron deficiency at specific carbon atoms. The pronounced electronegativity of fluorine atoms localized electron density in discrete molecular regions, creating an uneven dipole moment distribution, thereby resulting in a lower dielectric constant. The Estate_VSA4 (d_9_) demonstrated minimal contribution (1.18%), which quantifies the surface area contributions of atoms within an E-state index range (1.17–1.54), corresponding to regions of moderate polarity. The prevalence of atomic environments likely facilitated dielectric constant reduction by limiting excessive charge separation.

Descriptors BCUT2D_CHGHI (d_3_) and Chi2n (d_4_) quantify the balance between molecular topological complexity and local charge distribution. For **PI-a** and **PI-b**, BCUT2D_CHGHI (d_3_) and Chi2n (d_4_) values indicate a compromise between structural sophistication and charge delocalization. This balanced molecular architecture mitigates dynamic response constraints while maintaining sufficient polarization flexibility under external electric fields. **PI-c** demonstrates excessive charge localization and elevated topological complexity, constraining polarization pathways and diminishing dielectric response. SMR_VSA5 (d_6_) quantifies surface area distribution based on molar refractivity and correlates with molecular volume, revealing critical structural distinctions. The SMR_VSA5 value for **PI-c** is notably high, reaching 24.19. This is due to the presence of bulky -CF_3_ groups, which restrict the mobility of molecular chain segments and increase spatial hindrance. As a result, its dielectric constant is reduced. FpDensityMorgan3 (d_2_) reflects functional group density derived from Morgan fingerprints. **PI-b** exhibits a higher FpDensityMorgan3 value (2.13) compared to **PI-a** (1.85), suggesting the incorporation of additional functional groups. However, its dielectric constant lines between that of **PI-a** and **PI-c** due to the absence of steric-restrictive groups and high polar surface area contributions. For **PI-c**, the low FpDensityMorgan3 (d_2_) value (1.64) highlights a trade-off: the introduction of -CF_3_ substituents diminishes the density of other polar functional groups. This reduction partially counteracts the high polarization implied by its elevated PEOE_VSA6 value (54.59), ultimately limiting dielectric enhancement.

Based on descriptor contributions, to design PIs with lower dielectric constants, two main strategies could be adopted. First, reducing the TPSA helps to control the spatial distribution of polar groups. Second, introducing bulky groups, such as trifluoromethyl and adamantly, serves multiple purposes. These groups can increase the molecular volume, regulate the molar refractive index surface area (SMR_VSA5 (d_6_)), and restrict the movement of molecular segments through steric hindrance. This, in turn, can suppress the ability of dipole rearrangement. In addition, weak polar groups are concentrated in non-conjugated regions to optimize the density of functional groups (FpDensityMorgan3 (d_2_)). Finally, it is necessary to balance the post-molecular topological complexity and local charge distribution, which can limit the polarization path and reduce the dielectric response.

**Figure 8:**
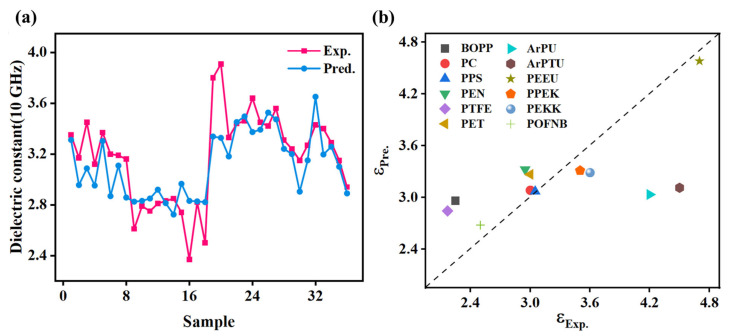
The application of machine learning models in distinct scenarios: (**a**) dielectric constant prediction of polyimide at 10 GHz, and (**b**) comparative dielectric constant prediction of various polymer materials at 1 kHz.

This study further demonstrates the expanded applicability of machine learning models in predicting frequency-dependent dielectric properties of polymers. It also evaluates their generalizability across diverse polymeric systems. As illustrated in Figure 8a, the GPR model shows limited predictive capability (R^2^ = 0.60, MAE = 0.17, RMSE = 0.22) for 36 PIs at 10 GHz [40], highlighting the challenges in cross-frequency extrapolation. This performance constraint stems from the model’s inability to capture complex polarization dynamics [24], including electronic, atomic, and orientational effect. These dynamics govern dielectric responses across broad frequency spectra. Future studies should focus on developing multi-frequency training datasets to better capture the spectrum of polarization mechanisms.

The model’s generalizability across polymer systems was systematically evaluated, as shown in Figure 8b. The GPR model demonstrated high predictive accuracy for PEEU, PC, PPS, and POFNB, but showed significant deviations for BOPP, PTFE, and PU. This highlights material-specific performance variations among the 12 polymers (structural details in Figure S1) [41,42]. Tanimoto similarity analysis between target polymers and PMDA-ODA (Table 2) revealed a correlation between structural similarity and prediction accuracy (e.g., PEEU = 0.32, PC = 0.15, PPS = 0.16). This indicates the model’s ability to capture essential PI structure features through domain-informed feature engineering. Based on these findings, optimizing PI-ML models should prioritize data augmentation using polymers with high structural similarity (Tanimoto similarity score > 0.15) or comparable polarity. For structurally dissimilar systems like BOPP and PTFE, cautious inclusion is recommended.

## 4. Conclusions

This work establishes a machine learning framework for accurately predicting polyimide dielectric constants, adding a new path for dielectric polymer material discovery. By integrating feature engineering and interpretable ML, the GPR model identified structural descriptors governing dielectric behavior, validated through experimental synthesis. The model’s high accuracy and interpretability underscore its utility in guiding PI design, particularly for reducing polar surface areas and optimizing charge distribution. However, the limited generalizability to dissimilar polymers and cross-frequency predictions highlights the necessity of augmenting datasets with multi-frequency measurements. Future work should focus on expanding descriptor diversity and incorporating dynamic polarization mechanisms to enhance predictive capabilities across broader material classes and frequency ranges. These insights facilitate the accelerated development of low-dielectric polymers tailored for electronic applications.

## Figures and Tables

**Table 1:** The ten selected descriptors and their definitions.

d_i_	Descriptors	Definitions
d_1_	MinEStateIndex	Minimum EState index
d_2_	FpDensityMorgan3	Morgan fingerprint, radius 3.
d_3_	BCUT2D_CHGHI	BCUT descriptors are a combination of descriptions based on the atomic number of each atom and the nominal bond types of adjacent and non-adjacent atoms.
d_4_	Chi2n	Similar to Hall Kier Chi2v, but uses nVal instead of valence.
d_5_	PEOE_VSA6	MOE Charge VSA Descriptor 6 (−0.10 ≤ x < −0.05)
d_6_	SMR_VSA5	MOE MR VSA Descriptor 5 (2.45 ≤ x < 2.75)
d_7_	SlogP_VSA8	MOE logP VSA Descriptor 8 (0.25 ≤ x < 0.30)
d_8_	TPSA	The polar surface area of a molecule based upon fragments.
d_9_	EState_VSA4	MOE logP VSA Descriptor 8 (0.72 ≤ x < 1.17)
d_10_	VSA_EState3	VSA EState Descriptor 3 (5.00 ≤ x < 5.41)

**Table 2:** Similarity comparison of polyimide with various polymers based on Tanimoto similarity scores.

Polymer	Tanimoto Similarity Score
BOPP	0.00
PC	0.15
PPS	0.16
PEN	0.15
PTFE	0.03
PET	0.12
ArPU	0.07
ArPTU	0.11
PEEU	0.32
PPEK	0.24
PEKK	0.21
POFNB	0.18

## Data Availability

Data will be made available on request.

## Supplementary Materials

The following supporting information can be downloaded at: https://www.mdpi.com/article/10.3390/polym17121622/s1.
