# Supplementary material for: Interpretable Machine Learning Prediction of Polyimide Dielectric Constants: A Feature-Engineered Approach with Experimental Validation
Source: Polymers (Basel). 2025 Jun 11;17(12):1622. doi: 10.3390/polym17121622 (PMC12197143; doi:10.3390/polym17121622)
Supplement: Supplementary file 1 [file polymers-17-01622-s001.zip › polymers-3685113-supplementary.pdf]

Interpretable Machine Learning Prediction of Polyimide Dielectric Constants: a Feature-Engineered Approach with Experimental Validation

Xiaojie He [1], Jiachen Wan [1], Songyang Zhang [1], Chenggang Zhang [1], Peng Xiao [4], Feng Zheng[1,*], Qinghua Lu [2,3,*]

[1]*School of Chemical Science and Engineering, Tongji University, Siping Road No. 1239, Shanghai, 200092, China*

[2]*China State Key Laboratory of Synergistic Chem-Bio Synthesis, School of Chemistry and Chemical Engineering, Shanghai Jiao Tong University, Shanghai 200240, China*

[3]*State Key Laboratory of Micro-Nano Engineering Science, Shanghai Jiao Tong University, 800 Dongchuan Road, Shanghai 200240, China*

[4]*Institute of Micro/Nano Materials and Devices, Ningbo University of Technology, Fenghua Road No. 201, Ningbo 315211 China*

Contents

NoteS1: Code for extracting descriptors from the polyimide molecular structure using RDKit.

NoteS2: This code for comparing the Tanimoto similarity between polyimide and other polymers.

Table S1: Reference links to literature on polyimide structural and property data.

Table S2: The feature importance scores of different descriptors and their respective meanings.

Table S3: Detailed calculation methods for dielectric-related molecular descriptors.

Table S4: Performance comparison of machine learning models for dielectric constant prediction.

NoteS3: The detailed synthesis process for three types of polyimides.

Table S5: Experimental and predicted ε values of polyimides.

Table S6: The values of the descriptors for the three polyimide structures and their contribution weights to the dielectric constant.

Figure S1: Typical structures of the 12 polymers.

NoteS1：Code for extracting descriptors from the polyimide molecular structure using RDKit.

```
from rdkit import Chem
from rdkit.Chem import Descriptors
from rdkit.ML.Descriptors import MoleculeDescriptors
import xlrd
import xlwt

input_excel_path = 'C:/Users/Administrator/Desktop/Die_data.xlsx'
excel_file = xlrd.open_workbook(input_excel_path)
sheet = excel_file.sheet_by_name("Sheet1")
smiles_column = sheet.col_values(0)

# Create Excel files to store descriptors and calculated results
output_desc_path = 'C:/Users/Administrator/Desktop/Descriptors.xlsx'
output_data_path = 'C:/Users/Administrator/Desktop/Des_data.xlsx'
desc_workbook = xlwt.Workbook(encoding='utf-8')
desc_sheet = desc_workbook.add_sheet("Descriptors_Info")
data_workbook = xlwt.Workbook(encoding='utf-8')
data_sheet = data_workbook.add_sheet('Descriptors_Data')

# Retrieve descriptor names and descriptions, and populate the descriptors sheet
descriptors = Descriptors._descList
descriptor_names = [desc[0] for desc in descriptors]
descriptor_descriptions = [desc[1] for desc in descriptors]

# Write descriptor names and descriptions to Descriptors_Info sheet
for i, (name, desc) in enumerate(zip(descriptor_names, descriptor_descriptions)):
    desc_sheet.write(i, 0, name)
    desc_sheet.write(i, 1, desc if isinstance(desc, (str, int, float)) else "N/A")

# Write descriptor names to the first row of Descriptors_Data sheet and add "SMILES" column
header
data_sheet.write(0, 0, "SMILES")
for i, name in enumerate(descriptor_names, start=1):
    data_sheet.write(0, i, name)

# Iterate through SMILES structures, compute descriptors, and populate the data sheet
calculator = MoleculeDescriptors.MolecularDescriptorCalculator(descriptor_names)
for row, smiles in enumerate(smiles_column, start=1):
    mol = Chem.MolFromSmiles(smiles)
    descriptors_values = calculator.CalcDescriptors(mol) if mol else [None] *
```

```
len(descriptor_names)

        data_sheet.write(row, 0, smiles)
        for col, value in enumerate(descriptors_values, start=1):
                data_sheet.write(row, col, value)

desc_workbook.save(output_desc_path)
data_workbook.save(output_data_path)
```

NoteS2：This code for comparing the Tanimoto similarity between polyimide and other polymers.

```
from rdkit import Chem, DataStructs
from rdkit.Chem import AllChem
import pandas as pd

def calculate_ecfp_similarity(reference_smiles, target_smiles_list):
        reference_mol = Chem.MolFromSmiles(reference_smiles)
        if reference_mol is None:
                print("Unable to generate reference molecule. Check SMILES format.")
                return

        reference_fp = AllChem.GetMorganFingerprintAsBitVect(reference_mol, 2, nBits=1024)
        similarity_scores = { }

        for target_smiles in target_smiles_list:
                target_mol = Chem.MolFromSmiles(target_smiles)
                if target_mol is None:
                        print(f"Invalid SMILES: {target_smiles}")
                        continue

                target_fp = AllChem.GetMorganFingerprintAsBitVect(target_mol, 2, nBits=1024)
                similarity = DataStructs.TanimotoSimilarity(reference_fp, target_fp)
                similarity_scores[target_smiles] = similarity

        return pd.DataFrame({
                'Target_SMILES': list(similarity_scores.keys()),
                'Tanimoto_Similarity': list(similarity_scores.values())
        })

# SMILES for the reference structure
reference_structure = '[nH]1c(=O)c2c(c1=O)cc1c(c2)c(=O)n(c1=O)c1ccc(cc1)Oc1ccccc1'
```

```
# SMILES list for target structures
target_structures = [
    'CC([H])C[H]',                    '[H]OC1=CC=C(C(C)(C2=CC=C(OC([H])=O)C=C2)C)C=C1',
'[H]C1=CC=C(S[H])C=C1',
    'O=C(O[H])C1=CC(C=CC(C(OCC[H])=O)=C2)=C2C=C1', '[H]C(F)(C([H])(F)F)F',
    'O=C(C1=CC=C(C(OCC[H])=O)C=C1)O[H]',
'[H]N(C([H])=O)C1=CC=C(CC2=CC=C(NC(NC3=CC=C(CC4=CC=C(N[H])C=C4)C=C3)=O)C
=C2)C=C1',
    'S=C(N[H])NC1=CC=C(CC2=CC=C([H])C=C2)C=C1',
'[H]OC1=CC=CC(OC2=CC=C(NC(NC3=CC=C([H])C=C3)=O)C=C2)=C1',

'[H]OC(C=C1)=CC=C1C(C2=C3C=CC=C2)=NN(C4=CC=C(C(C5=CC=C([H])C=C5)=O)C=C4
)C3=O',

'[H]OC1=CC=C(C(C2=CC=C(C(C3=CC=C(C(C)(C)OC4=CC=C(C(C5=CC(C(C6=CC=C([H])C
=C6)=O)=CC=C5)=O)C=C4)C=C3)=O)C=C2)=O)C=C1',

'O=C1N(C(C2C1C(/C([H])=C/C3=CC=CC=C3)OC2C([H])=C)=O)C4=CC(C(F)(F)F)=CC(C(F)(
F)F)=C4'
]

# Calculate similarity
result_df = calculate_ecfp_similarity(reference_structure, target_structures)
result_df.to_excel('C:/Users/Administrator/Desktop/similarity.xlsx', index=False)
print("Similarity results saved to C:/Users/Administrator/Desktop/similarity.xlsx.")
```

Table S1: Reference links to literature on polyimide structural and property data.

Reference ID	URL
1	https://doi.org/10.1016/j.eurpolymj.2019.109235
2	https://doi.org/10.1109/IPSS.1992.281979
3	https://doi.org/10.1021/acsami.8b07705
4	https://doi.org/10.1002/pola.26927
5	https://doi.org/10.1002/pola.27445
6	https://doi.org/10.1021/am502002v
7	https://doi.org/10.1039/D1PY00084E
8	https://doi.org/10.1002/macp.200350070
9	https://doi.org/10.1016/S0032-3861(02)00801-7
10	https://doi.org/10.1016/j.jfluchem.2010.03.008
11	https://doi.org/10.1007/s00396-003-1036-5
12	https://doi.org/10.1002/pi.3061
13	https://doi.org/10.1002/app.20532
14	https://doi.org/10.1002/pola.1217

15	https://doi.org/10.1002/pola.10748
16	https://doi.org/10.1016/j.eurpolymj.2004.01.006
17	http://dx.doi.org/10.1016/j.matchemphys.2014.01.039
18	https://doi.org/10.1002/pola.24748
19	https://doi.org/10.1002/pola.27007
20	https://doi.org/10.3144/expresspolymlett.2021.85
21	https://doi.org/10.1016/j.cplett.2021.139131
22	https://doi.org/10.1002/app.51972
23	https://doi.org/10.1016/j.polymer.2021.123963
24	https://doi.org/10.1080/10601325.2021.1952080
25	https://doi.org/10.1002/pol.20210112
26	https://doi.org/10.1016/j.jiec.2020.10.023
27	https://doi.org/10.1021/acsapm.0c01141
28	https://doi.org/10.1039/C7TC04220E
29	https://doi.org/10.1021/acs.macromol.9b00136
30	https://doi.org/10.1016/j.polymer.2009.10.022
31	https://doi.org/10.1007/s10118-021-2514-2
32	https://doi.org/10.1016/S0032-3861(98)00375-9
33	https://doi.org/10.1002/(SICI)1099-0518(19990401)37:7%3C937::AID-POLA10%3E3.0.CO;2-I
34	https://doi.org/10.1021/ma970520n
35	https://doi.org/10.1021/ma9706337
36	https://doi.org/10.1039/C7RA10493F
37	https://doi.org/10.3390/polym12071532
38	https://doi.org/10.1080/25740881.2020.1750651
39	https://doi.org/10.1080/10601325.2020.1739538
40	https://doi.org/10.1007/s10118-019-2225-0
41	https://doi.org/10.1016/j.eurpolymj.2018.09.029
42	https://doi.org/10.1021/acsomega.8b01364
43	http://dx.doi.org/10.1007/s11224-011-9865-1
44	https://doi.org/ 10.1039/d2py00062h
45	https://doi.org/10.1016/j.polymer.2005.05.034
46	https://doi.org/10.1016/j.polymer.2020.122963
47	https://doi.org/10.1039/C6RA00322B
48	https://doi.org/10.1002/masy.200350930
49	https://doi.org/10.1016/S0032-3861(99)00613-8
50	https://doi.org/10.1002/pat.1678
51	https://doi.org/10.1016/j.eurpolymj.2009.01.016
52	https://doi.org/10.1002/pola.23265
53	https://doi.org/10.1021/ma970930b
54	https://doi.org/10.1021/cm970341k
55	https://doi.org/10.1023/B:JPOL.0000021729.68546.07
56	http://dx.doi.org/10.1177/0954008311435798
57	http://dx.doi.org/10.1016/j.polymer.2013.08.014

58	http://dx.doi.org/10.1016/j.reactfunctpolym.2016.04.011
59	https://doi.org/10.1002/cjoc.201400587
60	https://doi.org/10.1002/pola.11091
61	https://doi.org/10.1007/s10118-020-2339-4
62	http://dx.doi.org/10.1016/j.eurpolymj.2008.01.041
63	http://dx.doi.org/10.1016/j.eurpolymj.2010.06.010
64	https://doi.org/10.3390/polym14214504
65	https://doi.org/10.1002/pola.27933
66	https://doi.org/10.1007/s00396-002-0798-5
67	https://doi.org/10.1002/pola.20116
68	https://doi.org/10.1007/s10965-006-9072-8
69	https://doi.org/10.1002/pi.4235
70	https://doi.org/10.1002/pola.23337
71	https://doi.org/10.1002/pola.11012
72	https://doi.org/10.1021/ma00091a028
73	https://doi.org/10.1016/S0032-3861(02)00359-2

Table S2: The feature importance scores of different descriptors and their respective meanings

Feature	Feature importance score	Description
BCUT2D_CHGHI	0.186200567	Implements BCUT descriptors From J. Chem. Inf. Comput. Sci., Vol. 39, No. 1, 1999
BCUT2D_MWLOW	0.124922237	Implements BCUT descriptors From J. Chem. Inf. Comput. Sci., Vol. 39, No. 1, 1999
BCUT2D_CHGLO	0.057815145	Implements BCUT descriptors From J. Chem. Inf. Comput. Sci., Vol. 39, No. 1, 1999
BCUT2D_LOGPHI	0.0541714	Implements BCUT descriptors From J. Chem. Inf. Comput. Sci., Vol. 39, No. 1, 1999
SlogP_VSA8	0.0276213	MOE logP VSA Descriptor 8 (0.25 <= x < 0.30)
EState_VSA4	0.027013228	EState VSA Descriptor 4 (0.72 <= x < 1.17)
BCUT2D_MRHI	0.026264192	Implements BCUT descriptors From J. Chem. Inf. Comput. Sci., Vol. 39, No. 1, 1999
BCUT2D_MRLOW	0.021630827	Implements BCUT descriptors From J. Chem. Inf. Comput. Sci., Vol. 39, No. 1, 1999

TPSA	0.021201039	The polar surface area of a molecule based upon fragments.
SMR_VSA5	0.019005424	MOE MR VSA Descriptor 5 (2.45 <= x < 2.75)
VSA_EState3	0.018983357	VSA EState Descriptor 3 (5.00 <= x < 5.41)
EState_VSA2	0.017907742	EState VSA Descriptor 2 (-0.39 <= x < 0.29)
BCUT2D_LOGPLOW	0.015888509	Implements BCUT descriptors From J. Chem. Inf. Comput. Sci., Vol. 39, No. 1, 1999
SlogP_VSA2	0.014343369	EState VSA Descriptor 2 (-0.39 <= x < 0.29)
EState_VSA3	0.014230145	EState VSA Descriptor 3 (0.29 <= x < 0.72)
FpDensityMorgan3	0.014226067	Morgan fingerprint, radius 3.
VSA_EState6	0.014001588	VSA EState Descriptor 6 (6.00 <= x < 6.07)
PEOE_VSA6	0.013924683	MOE Charge VSA Descriptor 6 (-0.10 <= x < -0.05)
FpDensityMorgan2	0.011294903	Morgan fingerprint, radius 2.
SlogP_VSA3	0.010387345	MOE logP VSA Descriptor 3 (-0.20 <= x < 0.00)
VSA_EState4	0.009957887	VSA EState Descriptor 4 (5.41 <= x < 5.74)
MinEStateIndex	0.009210516	Minimum EState index
SlogP_VSA6	0.008845013	MOE logP VSA Descriptor 6 (0.15 <= x < 0.20)
VSA_EState5	0.008693803	VSA EState Descriptor 5 (5.74 <= x < 6.00)
VSA_EState2	0.008596201	VSA EState Descriptor 2 (4.78 <= x < 5.00)
MaxAbsEStateIndex	0.008462713	Maximum absolute EState index
EState_VSA6	0.00845907	EState VSA Descriptor 6 (1.54 <= x < 1.81)
VSA_EState1	0.008287279	VSA EState Descriptor 1 (-inf < x < 4.78)
Chi2n	0.007815711	Similar to Hall Kier Chi2v, but uses nVal instead of valence.
SlogP_VSA4	0.007510801	MOE logP VSA Descriptor 4 (0.00 <= x < 0.10)
EState_VSA5	0.006939681	EState VSA Descriptor 5 (1.17 <= x < 1.54)
fr_para_hydroxylation	0.006821855	Number of para-hydroxylation sites
SMR_VSA9	0.006714952	MOE MR VSA Descriptor 9 (3.80 <= x <

		4.00)
PEOE_VSA7	0.006648507	MOE Charge VSA Descriptor 7 (-0.05 <= x < 0.00)
PEOE_VSA2	0.0066194	MOE Charge VSA Descriptor 2 (-0.30 <= x < -0.25)
PEOE_VSA9	0.006569766	MOE Charge VSA Descriptor 9 (0.05 <= x < 0.10)
MolLogP	0.006532726	Wildman-Crippen LogP value
Chi4n	0.00648349	Similar to Hall Kier Chi4v, but uses nVal instead of valence. This makes a big difference after we get out of the first row
BalabanJ	0.006457801	Calculate Balaban's J value for a molecule
PEOE_VSA8	0.006293587	MOE Charge VSA Descriptor 8 (0.00 <= x < 0.05)
EState_VSA7	0.005943529	EState VSA Descriptor 7 (1.81 <= x < 2.05)
BCUT2D_MWHI	0.00587705	Implements BCUT descriptors From J. Chem. Inf. Comput. Sci., Vol. 39, No. 1, 1999
Kappa3	0.005376143	Hall-Kier Kappa3 value
VSA_EState7	0.005339473	VSA EState Descriptor 7 (6.07 <= x < 6.45)
SMR_VSA7	0.005074207	MOE MR VSA Descriptor 7 (3.05 <= x < 3.63)
SlogP_VSA5	0.004754043	MOE logP VSA Descriptor 5 (0.10 <= x < 0.15)
SlogP_VSA1	0.004747808	MOE logP VSA Descriptor 1 (-inf < x < -0.40)
VSA_EState8	0.004474052	MOE logP VSA Descriptor 8 (0.25 <= x < 0.30)
HallKierAlpha	0.004174972	The Hall-Kier alpha value for a molecule
NumRotatableBonds	0.004015654	Number of Rotatable Bonds
qed	0.003775721	Calculate the weighted sum of ADS mapped properties
BertzCT	0.003773898	A topological index meant to quantify "complexity" of molecules.
Chi3n	0.003731849	Similar to Hall Kier Chi3v, but uses nVal instead of valence. This makes a big difference after we get out of the first row
EState_VSA10	0.003295089	EState VSA Descriptor 10 (9.17 <= x < 15.00)
Chi2v	0.003110614	From equations (5),(15) and (16) of Rev. Comp. Chem. vol 2, 367-422, (1991)
EState_VSA8	0.002705555	EState VSA Descriptor 8 (2.05 <= x <

		4.69)
SMR_VSA1	0.002685139	MOE MR VSA Descriptor 1 (-inf < x < 1.29)
Chi4v	0.002622524	From equations (5),(15) and (16) of Rev. Comp. Chem. vol 2, 367-422, (1991).
Chi1v	0.002517272	From equations (5),(11) and (12) of Rev. Comp. Chem. vol 2, 367-422, (1991)
PEOE_VSA10	0.002483296	MOE Charge VSA Descriptor 10 (0.10 <= x < 0.15)
SlogP_VSA10	0.002459168	MOE logP VSA Descriptor 10 (0.40 <= x < 0.50)
SMR_VSA10	0.002143741	MOE MR VSA Descriptor 10 (4.00 <= x < inf)
NumAliphaticRings	0.002009437	CalcNumAliphaticRings((Mol)mol) -> int : returns the number of aliphatic (containing at least one non-aromatic bond) rings for a molecule
Chi0	0.001955877	From equations (1),(9) and (10) of Rev. Comp. Chem. vol 2, 367-422, (1991)
Kappa2	0.001928991	Hall-Kier Kappa2 value
Chi3v	0.001920729	From equations (5),(15) and (16) of Rev. Comp. Chem. vol 2, 367-422, (1991)
Ipc	0.00186458	This returns the information content of the coefficients of the characteristic polynomial of the adjacency matrix of a hydrogen suppressed graph of a molecule.
MolMR	0.00174227	Wildman-Crippen MR value
SlogP_VSA11	0.001649774	MOE logP VSA Descriptor 11 (0.50 <= x < 0.60)
NumHeteroatoms	0.001642863	Number of Heteroatoms
fr_aryl_methyl	0.001631302	Number of aryl methyl sites for hydroxylation
fr_ether	0.001532383	Number of ether oxygens (including phenoxy)
Kappa1	0.00152747	Hall-Kier Kappa1 value
LabuteASA	0.001484426	Labute's Approximate Surface Area
Chi0n	0.001448103	Similar to Hall Kier Chi0v, but uses nVal instead of valence. This makes a big difference after we get out of the first row
SMR_VSA4	0.00143094	MOE MR VSA Descriptor 4 (2.24 <= x < 2.45)
EState_VSA9	0.001372203	EState VSA Descriptor 9 (4.69 <= x < 9.17)
EState_VSA1	0.001370665	EState VSA Descriptor 1 (-inf < x < -

		0.39)
NumAromaticRings	0.001368832	CalcNumAromaticRings((Mol)mol) -> int : returns the number of aromatic rings for a molecule
ExactMolWt	0.001245161	The exact molecular weight of the molecule
HeavyAtomMolWt	0.001234317	The average molecular weight of the molecule ignoring hydrogens
Chi1n	0.001203378	Similar to Hall Kier Chi1v, but uses nVal instead of valence
PEOE_VSA4	0.001176252	MOE Charge VSA Descriptor 4 (-0.20 <= x < -0.15)
NumAliphaticHeterocycles	0.001174308	CalcNumAliphaticHeterocycles((Mol)mol) -> int : returns the number of aliphatic (containing at least one non-aromatic bond) heterocycles for a molecule
VSA_EState9	0.00116662	VSA EState Descriptor 9 (7.00 <= x < 11.00)
NumValenceElectrons	0.001127658	The number of valence electrons the molecule has
Chi1	0.001004964	From equations (1),(11) and (12) of Rev. Comp. Chem. vol 2, 367-422, (1991)
SMR_VSA3	0.000995457	MOE MR VSA Descriptor 3 (1.82 <= x < 2.24)
PEOE_VSA11	0.000962847	MOE Charge VSA Descriptor 11 (0.15 <= x < 0.20)
PEOE_VSA1	0.00085207	MOE Charge VSA Descriptor 1 (-inf < x < -0.30)
NumHAcceptors	0.000803911	Number of Hydrogen Bond Acceptors
NumAliphaticCarbocycles	0.000755137	CalcNumAliphaticCarbocycles((Mol)mol) -> int : returns the number of aliphatic (containing at least one non-aromatic bond) carbocycles for a molecule
fr_bicyclic	0.000747503	Bicyclic
MolWt	0.000733722	The average molecular weight of the molecule
NOCount	0.000727478	Number of Nitrogens and Oxygens
PEOE_VSA3	0.000691502	MOE Charge VSA Descriptor 3 (-0.25 <= x < -0.20)
fr_Imine	0.000584223	Number of Imines
RingCount	0.000569982	Number of aromatic rings in the molecule
SMR_VSA2	0.000518167	MOE MR VSA Descriptor 2 (1.29 <= x < 1.82)
NumAromaticCarbocycles	0.00051447	CalcNumAromaticCarbocycles((Mol)mol) -> int : returns the number of aromatic

		carbocycles for a molecule
Chi0v	0.000487802	From equations (5),(9) and (10) of Rev. Comp. Chem. vol 2, 367-422, (1991)
SMR_VSA6	0.000481347	MOE MR VSA Descriptor 6 (2.75 <= x < 3.05)
EState_VSA11	0.000458138	EState VSA Descriptor 11 (15.00 <= x < inf)
fr_aniline	0.000386623	Number of anilines
fr_NH0	0.000360911	Number of Tertiary amines
HeavyAtomCount	0.000334753	Number of heavy atoms a molecule.
fr_halogen	0.000323051	Number of halogens
fr_nitrile	0.000290141	Number of nitriles
fr_unbrch_alkane	0.000250766	Number of unbranched alkanes of at least 4 members (excludes halogenated alkanes)
fr_C_O_noCOO	0.000203217	Number of carbonyl O, excluding COOH
SlogP_VSA12	0.000180777	MOE logP VSA Descriptor 12 (0.60 <= x < inf)
fr_ester	0.000175987	Number of esters
NumSaturatedRings	0.000124764	CalcNumSaturatedRings((Mol)mol) -> int : returns the number of saturated rings for a molecule
PEOE_VSA14	0.000119781	MOE Charge VSA Descriptor 14 (0.30 <= x < inf)
fr_Ar_N	0.000111131	Number of aromatic nitrogens
fr_sulfone	0.000110225	Number of sulfone groups
PEOE_VSA13	0.000107932	MOE Charge VSA Descriptor 13 (0.25 <= x < 0.30)
fr_NH1	9.60639E-05	Number of Secondary amines
NumHDonors	9.35239E-05	Number of Hydrogen Bond Donors
PEOE_VSA12	8.47646E-05	MOE Charge VSA Descriptor 12 (0.20 <= x < 0.25)
fr_allylic_oxid	8.40685E-05	Number of allylic oxidation sites excluding steroid dienone
fr_methoxy	7.83986E-05	Number of methoxy groups -OCH3
NumAromaticHeterocycles	6.76987E-05	CalcNumAromaticHeterocycles((Mol)mol) -> int : returns the number of aromatic heterocycles for a molecule
fr_pyridine	5.67981E-05	Number of pyridine rings
fr_ketone	5.53524E-05	Number of ketones
fr_alkyl_halide	5.0664E-05	Number of alkyl halides
fr_Nhpyrrole	4.20085E-05	Number of H-pyrrole nitrogens
VSA_EState10	2.4356E-05	VSA EState Descriptor 10 (11.00 <= x < inf)
fr_imide	1.47195E-05	Number of imide groups

fr_amide	8.79508E-06	Number of amides
NumSaturatedCarbocycles	7.59783E-06	CalcNumSaturatedCarbocycles((Mol)mol) -> int : returns the number of saturated carbocycles for a molecule
SlogP_VSA7	6.67532E-06	MOE logP VSA Descriptor 7 (0.20 <= x < 0.25)
NHOHCount	5.96453E-06	Number of NHs or OHs
NumSaturatedHeterocycles	4.57197E-06	CalcNumSaturatedHeterocycles((Mol)mol) -> int : returns the number of saturated heterocycles for a molecule
fr_N_O	3.80281E-06	Number of hydroxylamine groups
PEOE_VSA5	2.23555E-06	MOE Charge VSA Descriptor 5 (-0.15 <= x < -0.10)

Table S3: Detailed calculation methods for dielectric-related molecular descriptors.

Descriptor	Calculation Method	Reference
MinEStateIndex	$s_i = I_i + \sum_{j=1}^{A} \frac{I_i - I_j}{(d_{ij}+1)^k}$ Where s_i represents the E-State Index value of atom i, I_i is the intrinsic state value of atom i, d_{ij} denotes the topological distance between atoms i and j, and k is an adjustment factor used to control the rate of distance attenuation.	[1]
Chi2n	$\mathrm{Chi2n} = \sum_{(i,j)\in second\ neighbors} \frac{1}{\sqrt{\left((d_i \cdot d_j)\cdot(v_i \cdot v_j)\cdot(e_i \cdot e_j)\cdot b_{ij}\right)}}$ In this equation, d_i and d_j represent the degree of atoms i and j, v_i and v_j denote their valency, e_i and e_j represent their electronegativity, and b_{ij} is the bond type factor between atoms i and j (assigned as 1 for single bonds, 1.5 for double bonds, and 2 for triple bonds).	[2]
BCUT2D_CHGHI	$\begin{bmatrix} q_i & bond_{ij}\cdot\sqrt{q_i \cdot q_j} & 0 \\ bond_{ij}\cdot\sqrt{q_i \cdot q_j} & q_j & bond_{jk}\cdot\sqrt{q_j \cdot q_k} \\ 0 & bond_{jk}\cdot\sqrt{q_j \cdot q_k} & q_k \end{bmatrix}_{\lambda_{max}}$ In a molecule, q_i represents the attribute value of the i-th atom, usually referred to as the partial charge. By combining the bond weight $bond_{ij}$ and the partial charges of atoms q_i and q_j, a	[3]

	weighted bond expression $bond_{ij} \times q_i \cdot q_j$ can be constructed. This formula comprehensively describes the charge interaction between two atoms and the strength of their connection. Subsequently, a weighted matrix is built, and through eigenvalue decomposition, the largest eigenvalue of the matrix is taken as the value of the descriptor.	
SlogP_VSA8	$\sum^{N}_{\substack{i=1 \\ -0.4 \le logP_i < -0.2}} VSA_i \quad \log P_i = i \sum a_i n_i$	[4]

Table S4: Performance comparison of machine learning models for dielectric constant prediction.

Model	Train			Test		
	R^2	RMSE	MAE	R^2	RMSE	MAE
**XGBoost**	0.97	0.10	0.13	0.84	0.15	0.11
**GPR**	0.96	0.07	0.05	0.90	0.10	0.08
**ANN**	0.90	0.11	0.09	0.85	0.14	0.11
**RF**	0.89	0.11	0.08	0.83	0.15	0.13
**SVM**	0.87	0.13	0.10	0.82	0.16	0.12

NoteS3：The detailed synthesis process for three types of polyimides.

Take BTDA-DABA as an example. 50 ml of N,N dimethylacetamide (DMAc) was injected into a clean and nitrogen-filled three-necked flask, then 11.36 g (0.05 mol) of 4,4'-Diaminobenzanilide (DABA) was added to it and fully dissolved to become a clarified solution. 16.19 g (0.05025 mol) of 3,3',4,4'-benzophenonetetracarboxylic dianhydride (BTDA) was added to the solution in batches and slowly. After thorough stirring for 12 h a brown transparent viscous poly(amic acid (PAA) liquid was obtained. Afterwards, the PAA solution was uniformly coated onto clean and dry glass plates to form a liquid film of fixed thickness, and the solvent was removed in a vacuum oven at 100 ℃. The samples were then subjected to thermal imidization according to a temperature-controlled program of 100 ℃/1h, 150 ℃/1h, 200 ℃/1h, 300 ℃/1h and 330 ℃/2h to prepare the polyimide films. Following immersion in deionized water for a period of time, the film could be peeled off from the substrate. The samples with a thickness of 50-100 μm were prepared for further measurements of the dielectric properties.

Table S5: Experimental and predicted ε values of polyimides.

**PI**	**ε exp. (1kHz)**	**ε GPR pre. (1kHz)**	**Mean percentage deviation (%)**

**PI-a**	**4.11**	**4.01**	
**PI-b**	**3.28**	**3.30**	**2.24**
**PI-c**	**2.99**	**2.88**	

Table S6: The values of the descriptors for the three polyimide structures and their contribution weights to the dielectric constant.

**Descriptors**	**Contribution**			Sensitivity ($\times 10^{-5}$)	Normalized Importance (%)
	PI-a	PI-b	PI-c		
MinEStateIndex	-0.61	-0.57	-6.04	2.03	3.74
FpDensityMorgan3	1.85	2.13	1.64	5.22	9.62
BCUT2D_CHGHI	2.37	2.37	2.73	-4.59	8.47
Chi2n	8.95	9.12	10.32	-4.43	8.16
PEOE_VSA6	30.33	30.33	54.60	7.61	14.02
SMR_VSA5	0	0	24.19	-1.21	2.23
SlogP_VSA8	0	22.55	0	-6.02	11.11
TPSA	129.72	126.65	83.55	17.59	32.42
EState_VSA4	4.90	10.46	17.19	-6.40	1.18
VSA_EState3	4.94	2.19	1.83	4.92	9.07

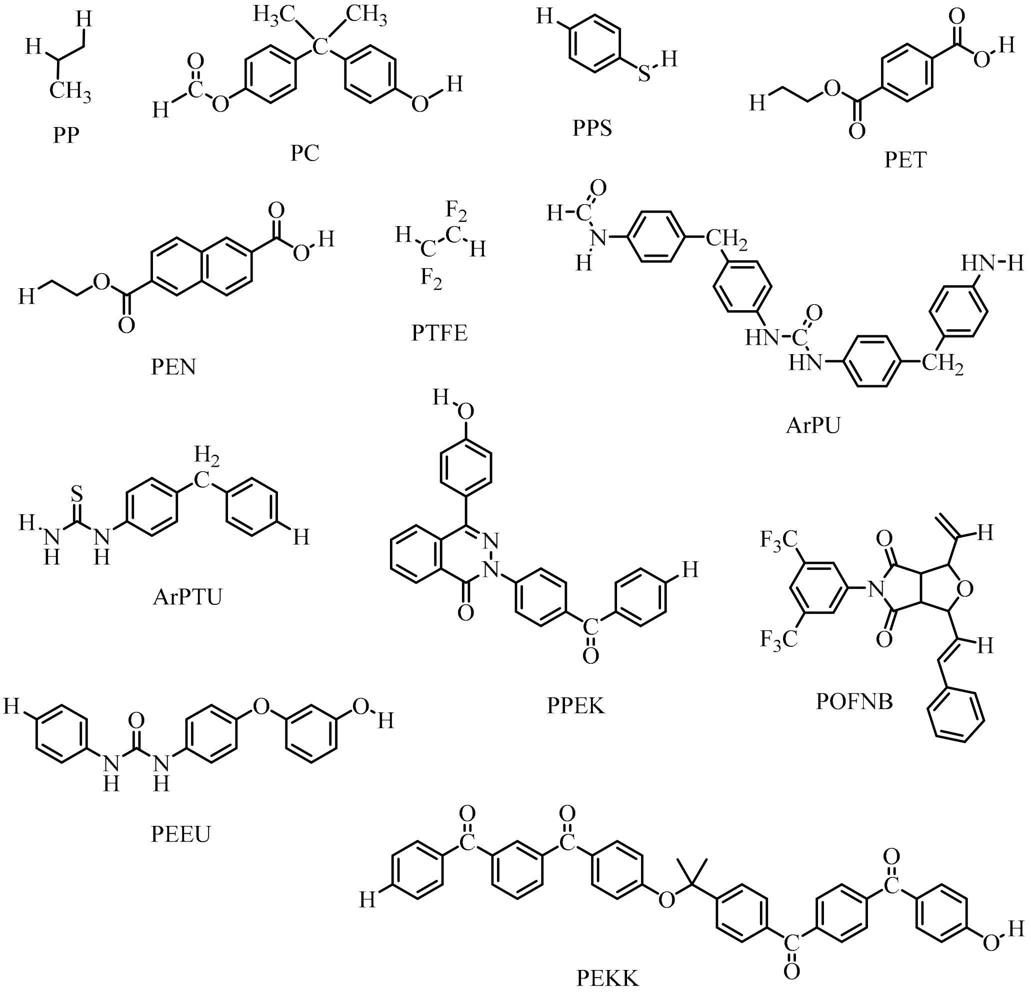

Figure S1: Typical structures of the 12 polymers.

References

[1] K. Roy, I. Mitra, Electrotopological state atom (E-state) index in drug design, QSAR, property prediction and toxicity assessment, Curr. comput.-aided Drug Design 8(2) (2012) 135-58.

[2] L.H. Hall, L.B. Kier, The Molecular Connectivity Chi Indexes and Kappa Shape Indexes in Structure-Property Modeling, Rev. Comput. Chem.1991, pp. 367-422.

[3] L. Cao, Z. Xu, T. Shang, C. Zhang, X. Wu, Y. Wu, S. Zhai, Z. Zhan, H. Duan, Multi_CycGT: A Deep Learning-Based Multimodal Model for Predicting the Membrane Permeability of Cyclic Peptides, J. Med. Chem. 67(3) (2024) 1888-1899.

[4] J. Yu, L. Zhang, Y. Zhang, W. An, Q. Guo, Y. Zhao, M. Yang, Functional recognition of structure-diverse odor molecules in drinking water based on QSOR study, Chemosphere 211 (2018) 371-378.
